# Supplementary material for: Causal Relationship between Aging and Anorexia Nervosa: A White-Matter-Microstructure-Mediated Mendelian Randomization Analysis
Source: Biomedicines. 2024 Aug 16;12(8):1874. doi: 10.3390/biomedicines12081874 (PMC11351342; doi:10.3390/biomedicines12081874)
Supplement: Supplementary file 1 [file biomedicines-12-01874-s001.zip › Supplementary Figure/Figure S8/Leaveoneout plot of MR analysis for TL to ubm-b-1483.pdf]

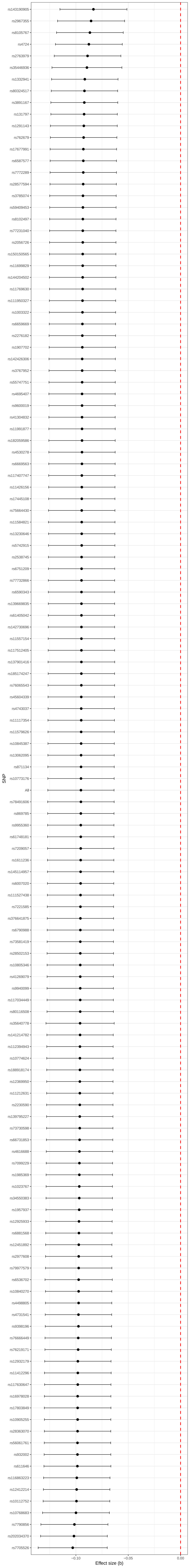

rs143190905
rs2967355
rs8105767
rs4724
rs2763979
rs35446936
rs1332941
rs80324517
rs3891167
rs131797
rs1291143
rs762679
rs17677991
rs6587577
rs7772289
rs28577594
rs3785074
rs59409453
rs8102497
rs77231040
rs2056726
rs150150565
rs11699829
rs144204502
rs11769630
rs111950327
rs1003322
rs6659669
rs2276182
rs1907702
rs142426306
rs3767952
rs55747751
rs4695407
rs9600019
rs41304832
rs11991877
rs182059586
rs4530278
rs6669563
rs117407747
rs11426156
rs17445108
rs75664430
rs11584821
rs13230646
rs5742915
rs2538745
rs6751209
rs77732866
rs6590343
rs139669835
rs61405042
rs142730696
rs11557154
rs117512405
rs137901416
rs185174247
rs76065543
rs45604339
rs4743037
rs11117354
rs11579626
rs10845387
rs13062095
rs871134
rs10773176
A8
rs78491606
rs869785
rs9955360
rs61748181
rs7209057
rs1611236
rs145114957
rs6007020
rs111527438
rs7221585
rs376641875
rs6790988
rs73581419
rs28502153
rs10805346
rs41269079
rs9940099
rs117034449
rs80116508
rs35640778
rs141214782
rs112394943
rs10774624
rs188918174
rs12369950
rs11212631
rs2230590
rs139795227
rs73730598
rs66731853
rs4616688
rs7099229
rs1985369
rs1023767
rs34550383
rs1957937
rs12925933
rs6881568
rs12451892
rs2977608
rs79977579
rs6536702
rs10840270
rs4498805
rs4731541
rs9398196
rs76666449
rs76219171
rs12932179
rs11412296
rs117630647
rs16978028
rs17803849
rs10905255
rs28363070
rs56061761
rs932002
rs611646
rs116863223
rs12412214
rs10112752
rs10768683
rs7790856
rs202034370
rs7705526
SNP
−0.10
−0.05
0.00
Effect size (b)
